# Implications of child poverty reduction targets for public health and health inequalities in England: a modelling study between 2024 and 2033

**DOI:** 10.1136/jech-2024-222313

**Published:** 2024-08-25

**Authors:** Ronan McCabe, Roxana Pollack, Philip Broadbent, Rachel M Thomson, Erik Igelström, Anna Pearce, Clare Bambra, Davara Lee Bennett, Alexiou Alexandros, Konstantinos Daras, David Taylor-Robinson, Benjamin Barr, Srinivasa Vittal Katikireddi

**Affiliations:** 1 MRC/CSO Social & Public Health Sciences Unit, University of Glasgow, Glasgow, UK; 2 Population Health Sciences Institute, Newcastle University Institute for Health and Society, Newcastle upon Tyne, UK; 3 Public Health, Policy & Systems, University of Liverpool, Liverpool, UK

## Abstract

**Background:**

We investigated the potential impacts of child poverty (CP) reduction scenarios on population health and health inequalities in England between 2024 and 2033.

**Methods:**

We combined aggregate local authority-level data with published and newly created estimates on the association between CP and the rate per 100 000 of infant mortality, children (aged <16) looked after, child (aged <16) hospitalisations for nutritional anaemia and child (aged <16) all-cause emergency hospital admissions. We modelled relative, absolute (per 100 000) and total (per total population) annual changes for these outcomes under three CP reduction scenarios between 2024 and 2033—*low-ambition* (15% reduction), *medium-ambition* (25% reduction) and *high-ambition* (35% reduction)—compared with a baseline CP scenario (15% increase). Annual changes were aggregated between 2024 and 2033 at national, regional and deprivation (IMD tertiles) levels to investigate inequalities.

**Results:**

All CP reduction scenarios would result in substantial improvements to child health. Meeting the *high-ambition* reduction would decrease total cases of infant mortality (293; 95% CI 118 to 461), children looked after (4696; 95% CI 1987 to 7593), nutritional anaemia (458, 95% CI 336 to 574) and emergency admissions (32 650; 95% CI 4022 to 61 126) between 2024 and 2033. Northern regions (eg, North East) exhibited the greatest relative and absolute benefit. The most deprived tertile would experience the largest relative, absolute and total benefit; under *high-ambition* reduction, total infant mortality cases were predicted to fall by 126 (95% CI 51 to 199) in the most deprived tertile compared with 71 (95% CI 29 to 112) in the least between 2024 and 2033.

**Conclusions:**

Achieving reductions in CP could substantially improve child health and reduce health inequalities in England.

## Background

Child poverty is a key determinant of population health and health inequalities.^1^ Experiencing child poverty is associated with worse outcomes across a wide range of early years health indicators, with evidence suggesting that these associations are often causal.^2–4^ Child poverty also likely reinforces the clustering and accumulation of adverse exposures.^5^ Government policy exerts a major influence over rates of child poverty. For example, higher levels of social spending were associated with lower levels of child poverty across European countries in the aftermath of the 2008 financial crisis, whereas countries such as the UK that have enacted high levels of austerity following the crisis—including retrenchment of social spending and local government budgets—have exhibited worse trends in child health outcomes.^6–9^

In the UK, progress had been made in reducing child poverty with the ‘New Labour’ Government (1997–2010) introducing several policies under the aim of being “(…) the first generation to end child poverty (in the UK)”.^10^ These included targeted measures to supplement income such as the Child Tax Credit and increases in Child Benefit, along-side other measures to improve early years services such as Sure Start programmes.^10 11^ Consequently, relative child poverty (before housing costs, BHC) declined from 27% to 20% across this period (a 25.9% reduction)^12^; corresponding declines in infant mortality rates were observed, particularly in the most deprived areas.^13^ However, following the 2008 financial crash and the subsequent enactment of austerity measures by consecutive Conservative-led Governments since 2010, child poverty levels began rising from 17% in 2014 to 23% (BHC) in 2020.^12^ This period coincided with a rise in infant mortality.^9^ Child poverty is responsive to policy; levels fell to 19% in 2021 following a brief uplift in social spending which was withdrawn by the end of that same year, with levels rising back to 22% in 2023.^12 14^ The UK also exhibits wide geographical variation in child poverty levels and its devolved governments have (although limited) powers to influence levels; for example, in 2021, the Scottish Government introduced the weekly Scottish Child Payment for low-income parents/carers, although the impact of this policy on child poverty has not been evaluated yet.^15 16^ The societal effects of the COVID-19 pandemic and the ongoing ‘cost of living’ crisis have heightened concerns about the level of child poverty in the UK and its current and future impact on child health.^17–21^ While some broad measures have been taken by the UK Government in response to this situation, there has been a lack of policy explicitly addressing rising child poverty—such as removing the ‘two-child limit’ and ‘benefit cap’ on financial support.^22^ Similarly, the UK Government’s initiative to ‘level up’ regional inequalities makes no reference to child poverty, despite the wide regional variations in child poverty rates.^16 23^ As such, it is important to understand how levels of child poverty could change under different hypothetical policy scenarios and the likely consequences these scenarios would have for child health.

We therefore aimed to investigate the potential impact of meeting different child poverty reduction scenarios on child health outcomes and inequalities in England over the next decade. We selected four child health outcomes which are associated with poverty and deprivation in childhood and for which there were local authority-level data available in England: (1) infant mortality; (2) children (<16 years old) entering local authority care; (3) child (<16 years old) hospital admissions for nutritional anaemia; and (4) child (<16 years old) all-cause emergency hospital admissions.^9 13 24–27^ While children entering care is not a direct measure of health, it is associated with a range of short-term and long-term adverse health consequences.^24^

## Methods

### Study setting and design

We created a dynamic policy simulation model using aggregated local authority-level data from England. This model allows for the exploration of ex-ante policy impacts under different scenarios between 2024 and 2033, drawing on existing data and published evidence of the relationship between child poverty and health outcomes.^28^

### Data

This ecological study used data for 145 English upper-tier local authorities (UTLAs). We excluded four UTLAs due to either small population size or irreconcilable boundary changes over the study period (City of London, Isles of Scilly, Bournemouth, Christchurch, and Poole and Dorset)^24^ and two further UTLAs due to a lack of published outcome data (Buckinghamshire and Northamptonshire). Exposure data on relative child poverty were acquired from the children in low-income families (CiLIF) statistics, compiled by the Department of Work and Pensions and His Majesty’s Revenue and Customs.^29^ Outcome data for infant mortality were derived from the Office for National Statistics (ONS).^30^ Data for looked-after children were obtained from the UK Government’s Department of Education,^31^ and local authority-level data on the number of hospitalisations for nutritional anaemia and all-cause emergency admissions were derived from NHS Hospital Episode Statistics data and supplied by the University of Liverpool’s Place-Based Longitudinal Data Resource (PLDR).^32^ Data on local authority-level income deprivation were derived from the 2019 Index of Multiple Deprivation (IMD), using the local authority average rank.^33^

### Exposure

We used the prevalence of relative child poverty BHC, captured in the CiLIF statistics, as our study exposure. This was defined as the proportion of children <16 years old living in families with an income of <60% of the contemporary national median income BHC. We used the 2020 CiLIF estimate to project annual values forward until the study end date in 2033 for each UTLA (see ‘modelled scenarios’ below); while estimates have subsequently been published until 2023, these are at present provisional.

### Outcomes

We examined four outcome measures at UTLA level: infant mortality, defined as the total number of deaths under the age of one per 100 000 live births per year; children looked after, defined as the total number of children (<16 years old) entering local authority care (whose care had been with local authorities for >24 hours period) per 100 000 of the <16 population per year; total child (<16 years old) hospitalisations for nutritional anaemia per 100 000 of the <16 population per year; and total child (<16 years old) all-cause emergency admissions per 100 000 of the <16 population per year. The final available values (numerator and denominator) for each outcome—2021 for infant mortality and children looked after, and 2019 for nutritional anaemias and emergency admissions—were held constant until start of the intervention period in 2024 (see online supplemental data).

### Data analysis

#### Effect estimates

We calculated additional cases attributable to changes in child poverty for each scenario using separate effect estimates for each outcome. For infant mortality and looked-after children, we used published estimates. For the former, we used an estimate from a time trends analysis of local authority-level data in England between 2000 and 2017, where a one-point change in the prevalence of child poverty was associated with a change in infant mortality of 5.8 (95% CI 2.4 to 8.9) deaths per 100 000 live births.^9^ For the latter, we used an estimate from a longitudinal ecological analysis of local authority-level data in England between 2015 and 2020, where a one-point change in the prevalence of child poverty was associated with a change in children looked after of 5.2 (95% CI 2.2 to 8.3) children per 100 000 children <16 years old.^24^

For nutritional anaemia and emergency admissions, we did not find relevant estimates in the published literature. Instead, we derived estimates for each outcome from new analysis of annual local authority-level data from the PLDR^32^ between 2015 and 2019. Estimates were derived using linear within-between regression analysis, in line with similar studies.^24^ This approach uses the strengths of both fixed and random effects models, integrating information on differences between and across areas. We found that a one-point change in the prevalence of child poverty was associated with 0.53 (95% CI 0.39 to 0.67) and 37.7 (95% CI 3.8 to 72.1) additional cases per 100 000 children <16 years old for nutritional anaemia and emergency admissions, respectively.

#### Modelled policy scenarios

We modelled a baseline child poverty scenario as a logarithmic annual increase (ie, curvilinear with a falling rate of change over time) from the 2020 prevalence of child poverty for each UTLA, resulting in a total cumulative increase of 15% from 2020 to 2033. This formed the baseline scenario to which the effects of other scenarios were compared (see below); that is, we were interested in modelling the potential effects of successful action to reduce child poverty versus unsuccessful or no action. Using the 2023 baseline prevalence of child poverty, we then modelled three scenarios at UTLA level over a 10-year period from 2024 until 2033 (see table 1): (1) *low ambition* reduction, a cumulative exponential decrease (ie, increasing rate of change over time) in child poverty of 15% on 2023 levels between 2027 and 2033 (3-year delay); (2) *medium ambition* reduction, a cumulative exponential decrease of 25% on 2023 levels between 2026 and 2033 (2-year delay); and (3) *high ambition* reduction, a cumulative exponential decrease of 35% on 2023 levels between 2025 and 2033 (1-year delay). We understood these scenarios to be realistic in light of the 26% fall in prevalence previously observed in the UK between 1997 and 2010 under previous governments.^34^ All scenarios were created using MS Excel (see online supplemental data).

#### Modelling approach

We calculated the annual number of attributable (avoided or added) cases at UTLA level for each outcome under each scenario: the annual relative change in child poverty (%) multiplied by the effect size per number exposed in that same year. We used a Monte Carlo approach to randomly sample (1000 iterations) from the distribution of the effect size of child poverty for each health outcome based on its mean and SE, taking the median of the sample to determine the point estimate of attributable cases, and the 2.5th and 97.5th percentiles for the upper and lower CIs. For each scenario compared with baseline, we report the change in cases for each outcome as the (1) total change per individuals exposed, (2) absolute change, as the risk difference (RD) per 100 000 exposed, and (3) relative change, as risk ratio (RR) at local authority level, regional level, national level and by IMD tertiles across the whole intervention period (2024–2033). Both RR and RD account for differences in population size and are thus suitable for comparison, but only compare extreme categories of the distribution. To quantify effects on inequalities in outcomes taking account for the whole distribution of deprivation, we estimated absolute and relative changes, respectively, as the difference in slope index of inequality (SII) and ratio of relative index of inequality (RII) under each scenario compared with baseline (see online supplemental appendix 1 for details).^35^ The SII can be interpreted as the difference in the rate of outcomes between the hypothetically most and least deprived local authorities, whereas the RII can be interpreted as the ratio between those local authorities.

## Findings

Across the 145 UTLAs included in analysis, the population-weighted mean prevalence of child poverty in 2023 projected under the baseline scenario was 20.7% (table 1). At regional level, the prevalence of child poverty was typically higher in northern regions compared with southern, with the North East having the highest median prevalence at 27.6% (IQR=4.2) and the South East and South West both had the lowest at 15.4% (IQR=8.5–6.7, respectively) in 2023. Across IMD tertiles, the median prevalence was 27.8% (IQR=10.1) in the most deprived tertile and 13.9% (IQR=4.6) in the least deprived tertile. Cases per 100 000 in 2023 are given for each outcome in table 2, with emergency admission being the most frequent and hospitalisations for nutritional anaemia being the least. For each outcome, cases tended to be highest in regions with high child poverty. Outcome trends (cases per 100 000 exposed) at national level over the period for which official data were available (2015–2019) are presented in online supplemental appendix 2 figure A: admissions fluctuated across this period although were rising 2017–2019, hospitalisations for nutritional anaemia continued rising, and infant mortality and children looked after both fell from 2017 onwards.

### Modelled changes

Increasingly ambitious scenarios corresponded to greater relative and absolute beneficial effects, with effect sizes in the high-ambition policy target around twice that of the low-ambition target across all outcome measures at all levels of aggregation (tables 2 and 3, online supplemental appendix 2 tables A,B).

Between 2024 and 2033 across England, compared with baseline, we anticipate a reduction in: infant mortality of 1.6% (293 avoided cases, 95% CI 118 to 461) under the high-ambition scenario versus 0.9% (155 avoided cases, 95% CI 62 to 244) under the low-ambition scenario; children looked after of 2% (4696 avoided cases, 95% CI 1987 to 7593) versus 1% (2483 avoided cases, 95% CI 1051 to 4015); hospitalisations for nutritional anaemia of 4.1% (458 avoided cases, 95% CI 336 to 574) versus 2.2% (242 avoided cases, 95% CI 177 to 304); and emergency admissions of 0.4% (32 650 avoided cases, 95% CI 4022 to 34 126) versus 0.2% (17 266 avoided cases, 95% CI 2127 to 32 324) (table 2 and online supplemental appendix 2 table A).

**Figure 1:**
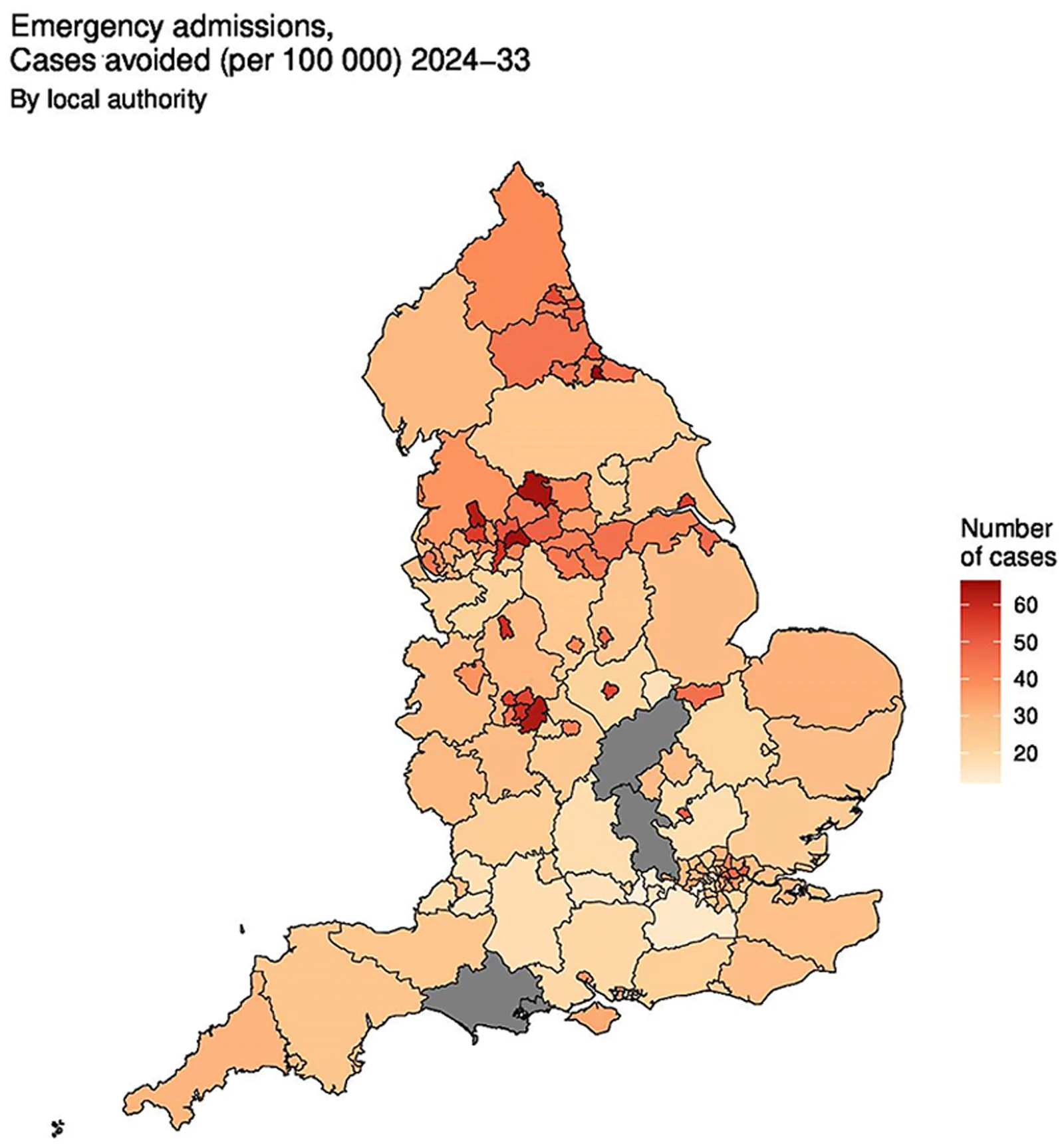
Absolute changes in avoided cases of emergency admissions (per 100 000) for the high ambition scenario (compared to baseline) at local authority level. Grey areas represent excluded local authorities.

**Figure 2:**
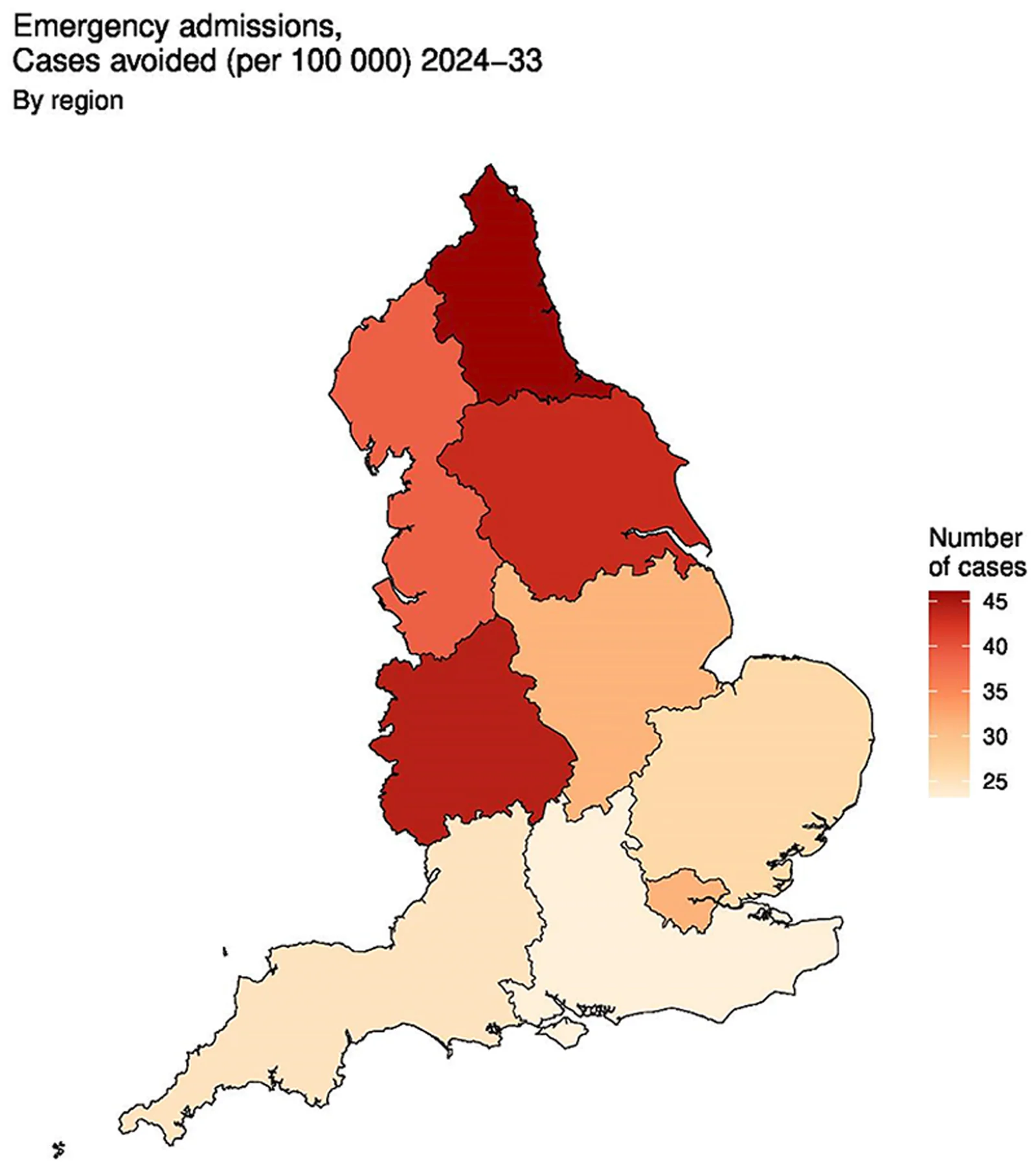
Absolute changes in avoided cases of emergency admissions (per 100 000) for the high ambition scenario (compared to baseline) at region level.

At regional level, estimated absolute reductions were typically higher in the north and west of England (eg, North East, West Midlands and Yorkshire and The Humber) compared with the south (see table 2); this pattern is highlighted in figures 1 and 2 for cases of emergency admissions avoided per 100 000 compared with baseline under the high-ambition scenario. Between 2024 and 2033, for all child poverty reduction scenarios, we anticipate cases avoided (compared with baseline) per 100 000 would be largest in the North East for all outcomes and smallest in the South East (table 2). Under the high-ambition scenario, estimated total avoided cases in the North East would be 18 (95% CI 7 to 28) for infant mortal, 298 (95% CI 126 to 482) for children looked after, 29 (95% CI 21 to 36) for nutritional anaemias, and 2070 (95% CI 255 to 3876) for emergency admissions (online supplemental appendix 2 table A). Regional patterns of relative change were less uniform (table 2), while total cases avoided were typically highest in regions with greater population size (eg, London) (online supplemental appendix 2 table A). At local authority level across reduction scenarios, absolute changes per 100 000 were highest in Middlesborough, Oldham, Bradford and Birmingham for all outcome measures (see online supplemental data); this is visually displayed for emergency admissions in figures 1 and 2.

**Figure 3:**
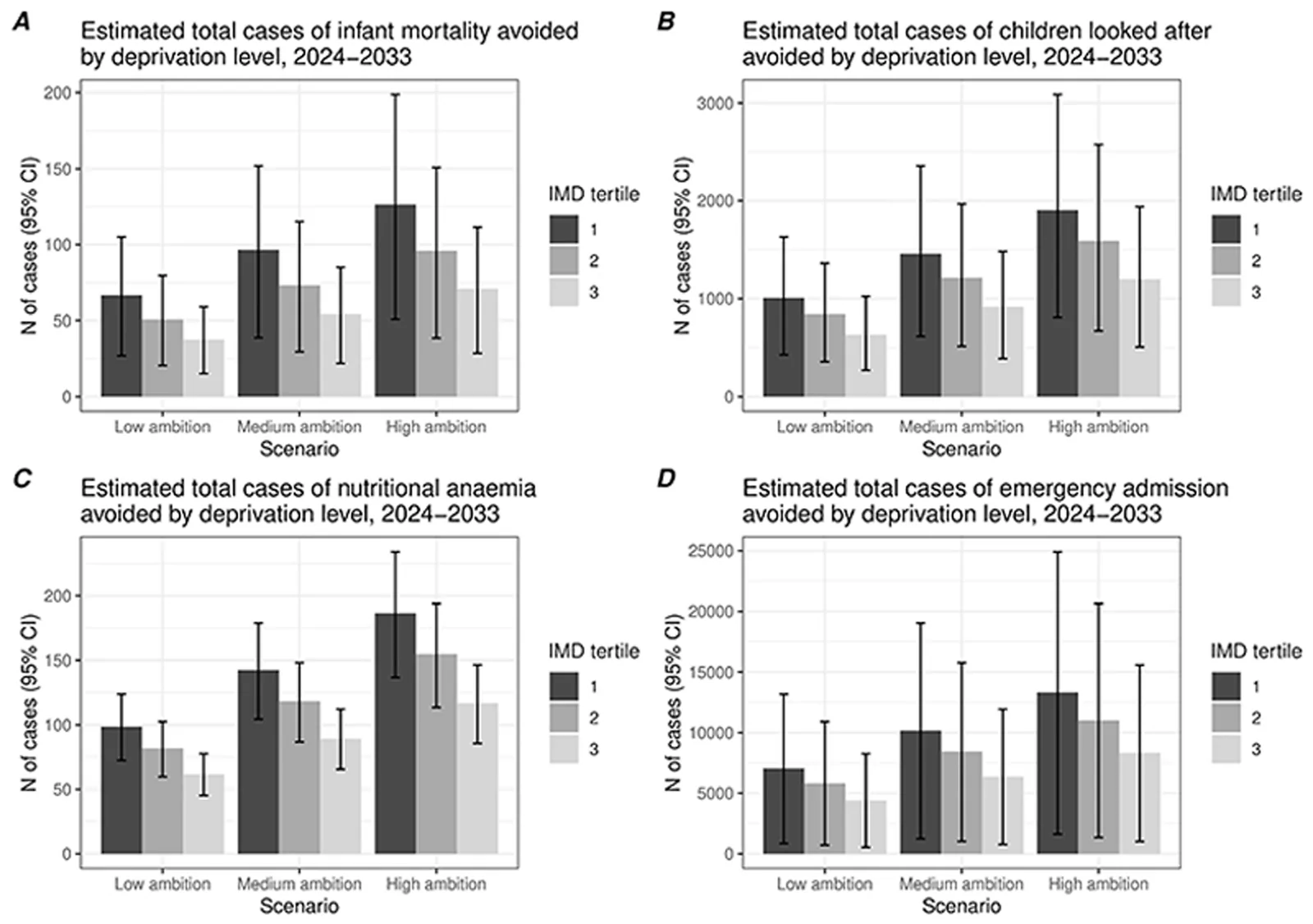
Estimated total avoided cases of four health outcomes under low, medium and high poverty reduction scenarios by Index of Multiple Deprivation (IMD tertile), 2024-2033.

Considering deprivation level, anticipated reductions on the difference scale (per 100 000) compared with baseline were highest in the most deprived tertile of UTLAs for all outcome measures (see table 3). Under the high-ambition scenario, this equated to a total avoided cases of 126 (95% CI 51 to 199) in the most deprived versus 71 (95% CI 29 to 112) in the least for infant mortality, 1907 (95% CI 807 to 3083) versus 1199 (95% CI 507 to 1939) for children looked after, 189 (95% CI 137 to 234) versus 117 (95% CI 86 to 146) for nutritional anaemias and 13 302 (95% CI 1639 to 24 903) versus 8 322 (95% CI 1025 to 15 581) for emergency admissions (online supplemental appendix 2 table B); total avoided cases under each scenario for each outcome measure are shown in figure 3. Changes on the ratio scale followed a broadly similar pattern (table 3). Greater reductions in child poverty were associated with greater reductions in absolute (SII difference) and relative (RII ratio) inequalities (table 3 and online supplemental appendix 2 table C, respectively).

## Discussion

Reducing child poverty will likely improve a range of child health outcomes and reduce health inequalities if similar or larger declines to those observed between 1997 and 2010 were achieved. We estimated relative, absolute and total changes in infant mortality, children looked after, nutritional anaemias and all-cause emergency admissions using local authority-level data in England under three different child poverty reduction scenarios between 2024 and 2033 compared with a baseline scenario of increasing child poverty. Achieving an ambitious but realistic reduction of 35% on 2023 levels would be expected to result in avoiding a total of 293 infant deaths, 4696 children entering care, 458 childhood admissions with nutritional anaemias and 32 650 childhood emergency admissions. These reductions would likely translate into significant savings for, and relieve pressure on, local authorities (in relation to children looked after) and health services. Benefits are likely to be greatest in the most disadvantaged areas, helping efforts to ‘level up’. Other health impacts that we have not been able to quantify are also likely.

We used administrative data from trusted sources and outcome estimates from previous empirical studies where available. Our modelling approach was simple and transparent, relying on a limited set of assumptions and a realistic baseline scenario (eg, we predicted mean relative child poverty BHC at 20.7%, whereas the provisional CiLif estimate for 2023 gives 20.1%).^29^ However, there are limitations to this work. We focused here on a limited set of outcomes which capture different dimensions of child health and for which there were data readily available. However, future work could extend this analysis to look at other common child health outcomes such as obesity and mental health which are both associated with child poverty.^36 37^ Relatedly, we used emergency admissions as a health outcome but acknowledge that they can be affected by health service access (changes in admission practice, transport, etc). Nonetheless, our analyses to parameterise the model excluded the COVID-19 pandemic when changes in practice were most likely to be problematic. We adopted the exposure of relative child poverty rate BHC. However, findings may have differed with alternative measures of child poverty such as absolute rates and rates after housing costs. Additionally, our analyses are predicated on the associations between child poverty and health outcomes accurately reflecting causal effects. While our analyses of changes within local authorities account for time-invariant confounding, risks of residual confounding remain. It is also possible that the effect estimates we observed for each outcome could differ as a consequence of the differing time periods for which data were available. Shorter time periods may lead to underestimated effect sizes within panel data analyses.^38^ This might imply our estimates of the impacts on emergency admissions and nutritional anaemia are underestimated. Relatedly, it is possible that the relationship between child poverty and outcomes does not exhibit the linear dose–response relationship that we have assumed here. A few local authorities were excluded due to small numbers, with possible consequences for overall estimates. Finally, our analyses are based on aggregate (ecological) data which could be subject to the ecological fallacy; although, while individual-level data analyses are of interest, these may be subject to the atomistic fallacy (ie, addressing child poverty could have positive impacts for communities beyond the individual).^39^ Aggregate data meant that we were also unable to account for variation within and between local authorities in the mechanisms influencing child poverty—for example, the depth of child poverty might differ and the health effects of addressing severe child poverty might differ from addressing less severe poverty. Furthermore, different policies to reduce child poverty (such as minimum wages, tax credits, welfare benefits) might have quite heterogenous effects that we do not distinguish. We would anticipate the impacts of the above factors to result in our estimates being conservative.

To our knowledge, this study is the first to explore the potential impacts of future child poverty reductions on a range of child health outcomes in England. It builds on previous empirical work that has highlighted the consequences of child poverty on outcomes such as infant mortality and children looked after in England.^9 13 24^ For example, this research found that reductions in child poverty in the UK between 1997 and 2010 led to a reduction in infant mortality, while subsequent increases in child poverty led to increases in infant mortality.^9 13^ Tying into factors influencing child poverty, previous studies have also found associations between increased local authority spending in England and reductions in hospital admissions for nutritional anaemia, although this association lacked precision among those <14 years old (rate ratio=0.97, 95% CI 0.90 to 1.05).^40^ Similarly, a study using local authority data by the Nuffield Trust showed that, in 2015/2016, the number of emergency admissions was higher with increasing deprivation among those <14 years old.^26^

We highlight that if policy-makers were to set and achieve child poverty targets for England—for example, through suggested measures such as removing the two-child limit and benefit cap^22^—this would likely improve child health, particularly among the most socioeconomically disadvantaged and ‘level up’ regional inequalities.

## Supplemental material

This content has been supplied by the author(s). It has not been vetted by BMJ Publishing Group Limited (BMJ) and may not have been peer-reviewed. Any opinions or recommendations discussed are solely those of the author(s) and are not endorsed by BMJ. BMJ disclaims all liability and responsibility arising from any reliance placed on the content. Where the content includes any translated material, BMJ does not warrant the accuracy and reliability of the translations (including but not limited to local regulations, clinical guidelines, terminology, drug names and drug dosages), and is not responsible for any error and/or omissions arising from translation and adaptation or otherwise.

## Figures and Tables

**Table 1:** Descriptive statistics for baseline exposure and outcomes, derived from modelled projections

Level	Population-weighted mean UTLA prevalence (%) of child poverty in 2023	Estimated cases per 100 000 in 2023			
		Infant mortality	Nutritional anaemias	Emergency admissions	Children looked after
*England*	*20.7*	321	11	7490	230
East Midlands	19.5	345	6	6644	218
East of England	16.3	274	7	6702	158
London	19.6	317	12	5188	196
North East	28.7	229	14	10 850	444
North West	24.2	324	18	10 615	308
South East	14.4	290	9	6870	190
South West	15.4	263	8	7998	210
West Midlands	27.5	465	13	8255	227
Yorkshire and the Humber	26.8	337	13	7466	275
1 (most deprived)	29.7	379	17	8177	305
2	20.7	306	10	7520	235
3 (least deprived)	13.9	288	8	6950	170

UTLA, upper tier local authority.

**Table 2:** Modelled relative and absolute changes (95% CI) under three child poverty reduction scenarios between 2024 and 2033, relative to a baseline scenario of increasing child poverty

Region	Relative change Risk ratio (RR), with 95% CI				Absolute change Risk difference (RD) per 100 000 exposed, with 95% CI		
	Low	Medium	High		Low	Medium	High
Infant mortality							
National level	0.991 (0.987 to 0.997)	0.988 (0.981 to 0.995)	0.984 (0.975 to 0.994)		−2.747 (−4.320 to −1.105)	−3.971 (−6.245 to −1.597)	−5.195 (−8.169 to −2.090)
East Midlands	0.993 (0.988 to 0.997)	0.989 (0.983 to 0.996)	0.986 (0.978 to 0.994)		−2.589 (−4.072 to −1.042)	−3.743 (−5.886 to −1.506)	−4.897 (−7.700 to −1.970)
East of England	0.992 (0.988 to 0.997)	0.989 (0.982 to 0.995)	0.985 (0.977 to 0.994)		−2.145 (−3.373 to −0.863)	−3.100 (−4.875 to −1.247)	−4.056 (−6.378 to −1.632)
London	0.992 (0.987 to 0.997)	0.988 (0.981 to 0.995)	0.984 (0.976 to 0.994)		−2.607 (−4.099 to −1.049)	−3.768 (−5.925 to −1.516)	−4.929 (−7.751 to −1.983)
North East	0.984 (0.976 to 0.994)	0.977 (0.965 to 0.991)	0.970 (0.954 to 0.988)		−3.780 (−5.945 to −1.521)	−5.464 (−8.593 to −2.198)	−7.148 (−1 1.241,−2.875)
NorthWest	0.990 (0.984 to 0.996)	0.986 (0.977 to 0.994)	0.981 (0.970 to 0.992)		−3.220 (−5.063 to −1.295)	−4.654 (−7.319 to −1.872)	−6.089 (−9.575 to −2.449)
South East	0.993 (0.990 to 0.997)	0.991 (0.985 to 0.996)	0.988 (0.981 to 0.995)		−1.903 (−2.993 to −0.766)	−2.751 (−4.326 to −1.107)	−3.599 (−5.660 to −1.448)
SouthWest	0.992 (0.988 to 0.997)	0.989 (0.983 to 0.996)	0.985 (0.977 to 0.994)		−2.023 (−3.182 to −0.814)	−2.925 (−4.599 to −1.177)	−3.826 (−6.017 to −1.539)
West Midlands	0.992 (0.988 to 0.997)	0.989 (0.982 to 0.995)	0.985 (0.977 to 0.994)		−3.663 (−5.760 to −1.473)	−5.295 (−8.326 to −2.130)	−6.927 (−10.892,−2.786)
Yorkshire and The Humber	0.990 (0.984 to 0.996)	0.985 (0.976 to 0.994)	0.980 (0.969 to 0.992)		−3.540 (−5.566 to −1.424)	−5.116 (−8.046 to −2.058)	−6.693 (−10.525,−2.692)
Children looked after (ages <16)							
National level	0.990 (0.983 to 0.996)	0.985 (0.976 to 0.994)	0.980 (0.969 to 0.992)		−2.380 (−3.849 to −1.007)	−3.441 (−5.564 to −1.456)	−4.502 (−7.279 to −1.905)
East Midlands	0.990 (0.983 to 0.996)	0.985 (0.976 to 0.994)	0.981 (0.969 to 0.992)		−2.240 (−3.622 to −0.948)	−3.238 (−5.236 to −1.370)	−4.236 (−6.850 to −1.793)
East of England	0.988 (0.981 to 0.995)	0.983 (0.972 to 0.993)	0.978 (0.964 to 0.991)		−1.875 (−3.032 to −0.793)	−2.710 (−4.382 to −1.147)	−3.545 (−5.733 to −1.500)
London	0.988 (0.980 to 0.995)	0.982 (0.971 to 0.993)	0.977 (0.963 to 0.990)		−2.255 (−3.646 to −0.954)	−3.260 (−5.271 to −1.379)	−4.264 (−6.895 to −1.805)
North East	0.993 (0.989 to 0.997)	0.990 (0.984 to 0.996)	0.987 (0.979 to 0.994)		−3.301 (−5.338 to −1.397)	−4.772 (−7.716 to −2.019)	−6.242 (−10.094,−2.642)
NorthWest	0.991 (0.985 to 0.996)	0.987 (0.979 to 0.994)	0.983 (0.972 to 0.993)		−2.783 (−4.500 to −1.178)	−4.023 (−6.504 to −1.702)	−5.262 (−8.509 to −2.227)
South East	0.991 (0.986 to 0.996)	0.987 (0.980 to 0.995)	0.984 (0.973 to 0.993)		−1.660 (−2.685 to −0.703)	−2.400 (−3.881 to −1.016)	−3.140 (−5.077 to −1.329)
SouthWest	0.992 (0.986 to 0.996)	0.988 (0.980 to 0.995)	0.984 (0.974 to 0.993)		−1.775 (−2.870 to −0.751)	−2.566 (−4.149 to −1.086)	−3.356 (−5.427 to −1.420)
West Midlands	0.986 (0.978 to 0.994)	0.980 (0.968 to 0.991)	0.974 (0.958 to 0.989)		−3.166 (−5.120 to −1.340)	−4.577 (−7.401 to −1.937)	−5.987 (−9.681 to −2.534)
Yorkshire and The Humber	0.989 (0.982 to 0.995)	0.984 (0.974 to 0.993)	0.979 (0.966 to 0.991)		−3.091 (−4.999 to −1.308)	−4.469 (−7.226 to −1.891)	−5.846 (−9.453 to −2.474)
Diagnoses of nutritional anaemia (ages <16)							
National level	0.978 (0.973 to 0.984)	0.968 (0.961 to 0.977)	0.959 (0.948 to 0.970)		−0.247 (−0.309 to −0.181)	−0.357 (−0.447 to −0.261)	−0.466 (−0.585 to −0.342)
East Midlands	0.961 (0.951 to 0.971)	0.943 (0.929 to 0.958)	0.926 (0.907 to 0.945)		−0.232 (−0.291 to −0.170)	−0.336 (−0.421 to −0.246)	−0.439 (−0.551 to −0.322)
East of England	0.974 (0.968 to 0.981)	0.963 (0.953 to 0.973)	0.951 (0.939 to 0.964)		−0.194 (−0.244 to −0.142)	−0.281 (−0.352 to −0.206)	−0.367 (−0.461 to −0.269)
London	0.979 (0.974 to 0.985)	0.970 (0.962 to 0.978)	0.961 (0.951 to 0.971)		−0.234 (−0.293 to −0.171)	−0.338 (−0.424 to −0.247)	−0.442 (−0.554 to −0.324)
North East	0.978 (0.972 to 0.984)	0.968 (0.959 to 0.976)	0.958 (0.947 to 0.969)		−0.342 (−0.429 to −0.251)	−0.494 (−0.620 to −0.362)	−0.647 (−0.811 to −0.474)
NorthWest	0.984 (0.980 to 0.988)	0.977 (0.972 to 0.983)	0.970 (0.963 to 0.978)		−0.288 (−0.362 to −0.211)	−0.417 (−0.523 to −0.306)	−0.545 (−0.684 to −0.400)
South East	0.981 (0.976 to 0.986)	0.973 (0.966 to 0.980)	0.964 (0.955 to 0.974)		−0.172 (−0.216 to −0.126)	−0.249 (−0.312 to −0.182)	−0.325 (−0.408 to −0.238)
SouthWest	0.978 (0.972 to 0.984)	0.968 (0.959 to 0.976)	0.958 (0.947 to 0.969)		−0.184 (−0.231 to −0.135)	−0.266 (−0.333 to −0.195)	−0.348 (−0.436 to −0.255)
West Midlands	0.974 (0.968 to 0.981)	0.963 (0.954 to 0.973)	0.952 (0.940 to 0.965)		−0.328 (−0.412 to −0.240)	−0.474 (−0.595 to −0.348)	−0.620 (−0.778 to −0.455)
Yorkshire and The Humber	0.975 (0.968 to 0.981)	0.963 (0.954 to 0.973)	0.952 (0.940 to 0.965)		−0.320 (−0.402 to −0.235)	−0.463 (−0.581 to −0.339)	−0.606 (−0.760 to −0.444)
Emergency admissions (ages <16)							
National level	0.998 (0.996 to 1.000)	0.997 (0.994 to 1.000)	0.996 (0.992 to 0.999)		−17.584 (−32.921 to 2.166)	−25.418 (−47.587 to −3.131)	−33.252 (−62.253 to −4.096)
East Midlands	0.998 (0.995 to 1.000)	0.996 (0.993 to 1.000)	0.995 (0.991 to 0.999)		−16.554 (−30.992 to 2.039)	−23.929 (−44.800 to −2.948)	−31.304 (−58.607 to −3.856)
East of England	0.998 (0.996 to 1.000)	0.997 (0.994 to 1.000)	0.996 (0.993 to 1.000)		−13.837 (−25.905 to 1.705)	−20.002 (−37.446 to −2.464)	−26.166 (−48.987 to −3.223)
London	0.997 (0.994 to 1.000)	0.995 (0.991 to 0.999)	0.993 (0.988 to 0.999)		−16.650 (−31.172 to 2.051)	−24.068 (−45.060 to −2.965)	−31.486 (−58.947 to −3.879)
North East	0.998 (0.996 to 1.000)	0.997 (0.994 to 1.000)	0.996 (0.992 to 0.999)		−24.374 (−45.633 to 3.003)	−35.233 (−65.962 to −4.340)	−46.092 (−86.291 to −5.678)
NorthWest	0.998 (0.996 to 1.000)	0.997 (0.995 to 1.000)	0.996 (0.993 to 1.000)		−20.556 (−38.485 to 2.532)	−29.714 (−55.631 to −3.661)	−38.872 (−72.776 to −4.789)
South East	0.998 (0.997 to 1.000)	0.997 (0.995 to 1.000)	0.997 (0.994 to 1.000)		−12.262 (−22.957 to 1.511)	−17.725 (−33.184 to −2.184)	−23.188 (−43.411 to −2.857)
SouthWest	0.998 (0.997 to 1.000)	0.998 (0.996 to 1.000)	0.997 (0.994 to 1.000)		−13.103 (−24.530 to 1.614)	−18.940 (−35.459 to −2.333)	−24.777 (−46.387 to −3.052)
West Midlands	0.997 (0.995 to 1.000)	0.996 (0.992 to 0.999)	0.995 (0.990 to 0.999)		−23.390 (−43.790 to 2.881)	−33.810 (−63.299 to −4.165)	−44.231 (−82.807 to −5.449)
Yorkshire and The Humber	0.997 (0.994 to 1.000)	0.996 (0.992 to 0.999)	0.994 (0.989 to 0.999)		−22.828 (−42.738 to 2.812)	−32.998 (−61.778 to −4.065)	−43.168 (−80.818 to −5.318)

Total changes per population exposed are presented in online supplemental appendix 2 table 2A.

**Table 3:** Modelled relative and absolute changes by Index of Multiple Deprivation (IMD) tertile and change in Slope Index of Inequality (SII) under three child poverty reduction scenarios between 2024 and 2033, relative to a baseline scenario of increasing child poverty

IMD tertile	Relative change Risk ratio (RR), with 95% CI				Absolute change Risk difference (RD) per 100 000 exposed, with 95% CI		
	Low	Medium	High		Low	Medium	High
Infant mortality							
1 (most deprived)	0.990 (0.984 to 0.996)	0.985 (0.977 to 0.994)	0.981 (0.970 to 0.992)		−3.878 (−6.098 to −1.560)	−5.605 (−8.814 to −2.255)	−7.333 (-11.531,−2.950)
2	0.991 (0.986 to 0.996)	0.987 (0.980 to 0.995)	0.983 (0.974 to 0.993)		−2.721 (−4.279 to −1.095)	−3.933 (−6.185 to −1.582)	−5.145 (−8.091 to −2.070)
3 (least deprived)	0.994 (0.990 to 0.997)	0.991 (0.986 to 0.996)	0.988 (0.981 to 0.995)		−1.824 (−2.869 to −0.734)	−2.637 (−4.147 to −1.061)	−3.450 (−5.425 to −1.388)
Children looked after							
1 (most deprived)	0.989 (0.982 to 0.995)	0.984 (0.974 to 0.993)	0.979 (0.966 to 0.991)		−3.425 (−5.539 to −1.450)	−4.951 (−8.007 to −2.095)	−6.478 (-10.474 to −2.741)
2	0.990 (0.984 to 0.996)	0.985 (0.976 to 0.994)	0.981 (0.969 to 0.992)		−2.385 (−3.856 to −1.009)	−3.447 (−5.574 to −1.459)	−4.510 (−7.292 to −1.908)
3 (least deprived)	0.991 (0.985 to 0.996)	0.986 (0.978 to 0.994)	0.982 (0.971 to 0.992)		−1.600 (−2.588 to −0.677)	−2.313 (−3.740 to −0.979)	−3.026 (−4.893 to −1.281)
Nutritional anaemias (ages <16)							
1 (most deprived)	0.979 (0.974 to 0.985)	0.970 (0.963 to 0.978)	0.961 (0.951 to 0.971)		−0.355 (−0.445 to −0.260)	−0.513 (−0.643 to −0.376)	−0.671 (−0.841 to −0.491)
2	0.975 (0.969 to 0.982)	0.964 (0.955 to 0.974)	0.953 (0.942 to 0.966)		−0.247 (−0.310 to −0.181)	−0.357 (−0.448 to −0.262)	−0.467 (−0.586 to −0.343)
3 (least deprived)	0.979 (0.974 to 0.985)	0.970 (0.962 to 0.978)	0.961 (0.951 to 0.971)		−0.166 (−0.208 to −0.121)	−0.240 (−0.300 to −0.176)	−0.313 (−0.393 to −0.230)
Emergency admissions (ages < :16)							
1 (most deprived)	0.997 (0.994 to 1.000)	0.996 (0.992 to 0.999)	0.994 (0.989 to 0.999)		−25.278 (−47.325 to 3.114)	−36.539 (−68.408 to −4.501)	−47.801 (−89.491 to −5.889)
2	0.998 (0.996 to 1.000)	0.997 (0.994 to 1.000)	0.996 (0.992 to 0.999)		−17.614 (−32.977 to 2.170)	−25.462 (−47.669 to −3.137)	−33.309 (−62.360 to −4.103)
3 (least deprived)	0.998 (0.997 to 1.000)	0.998 (0.995 to 1.000)	0.997 (0.994 to 1.000)		−11.811 (−22.113 to 1.455)	−17.073 (−31.965 to −2.103)	−22.336 (−41.816 to −2.752)
	**Change in SII** **Cases per 100 000 exposed, with 95% CI**						
Outcome	**Low**		**Medium**			**High**	
Infant mortality	−2.95 (−4.63 to −1.18)		−4.26 (−6.70 to −1.71)			−5.57 (−8.76 to −2.24)	
Children looked after	−2.57 (−4.15 to −1.09)		−3.71 (−6.00 to −1.57)			−4.85 (−7.84 to −2.05)	
Nutritional anaemias (ages <16)	−0.27 (−0.33 to −0.19)		−0.38 (−0.48 to −0.28)			−0.50 (−0.63 to −0.37)	
Emergency admissions (ages <16)	−18.96 (−35.49 to 2.34)		−27.40 (−51.30 to 3.38)			−35.84 (−67.11 to −4.42)	

Total changes by IMD tertile and changes in the Relative Index of Inequality (RII) are presented in online supplemental appendix table B,C, respectively.

## Data Availability

All data relevant to the study are included in the article or uploaded as supplementary information. Alternatively, the data is also available through the place-based longitudinal data resource: https://pldr.org/.

## What is Already Known on this Topic

⟹ Child poverty is a key determinant of population health and health inequalities.

## What this Study Adds

⟹ Child poverty is responsive to policy. We are the first to explore the health impact of meeting hypothetical future child poverty targets in England between 2024 and 2033. We show that reducing child poverty across this period would substantially improve child health and reduce health inequalities.

## How this Study Might Affect Research, Practice or Policy

⟹ We demonstrate the importance of renewed policy efforts to reduce child poverty.
