# Supplementary material for: Implications of child poverty reduction targets for public health and health inequalities in England: a modelling study between 2024 and 2033
Source: J Epidemiol Community Health. 2024 Aug 6;78(10):632–40. doi: 10.1136/jech-2024-222313 (PMC11420728; doi:10.1136/jech-2024-222313)
Supplement: Supplementary data [file jech-2024-222313supp002.pdf]

APPENDIX 1

The slope index of inequality (SII) and the relative index of inequality (RII) are regression-based metrics for quantifying the absolute and relative association, respectively, between a chosen health outcome and socio-economic rank.[1] Similar to the approach taken by Kypridemos et al. (2017)[2], we assigned a rank (between 0 and 1) to all 145 local authorities included in the study based on their individual Index of Multiple Deprivation (IMD) rank from the least to the most deprived, weighted by the population size for each outcome. We then estimated the linear association between this rank and the rate per 100 000 of each outcome under each scenario. We interpreted the slope of the regression line as the SII (i.e., the estimated difference in cases per 100 000 between the most and least deprived local authorities) and the sum of the slope and its intercept over its intercept – *(slope + intercept)/intercept* – as the RII (i.e., the ratio of estimated the difference in cases per 100 000 between the most and least deprived local authorities). To compare the SII and RII under each scenario to our baseline scenario of increasing child poverty, we took the difference in SIIs and the ratio of RIIs. The former was then interpreted as the estimated change in the absolute gap (cases per 100 000) between most and least deprived, while the latter was interpreted as the % change in the relative gap between most and least deprived.

APPENDIX 2

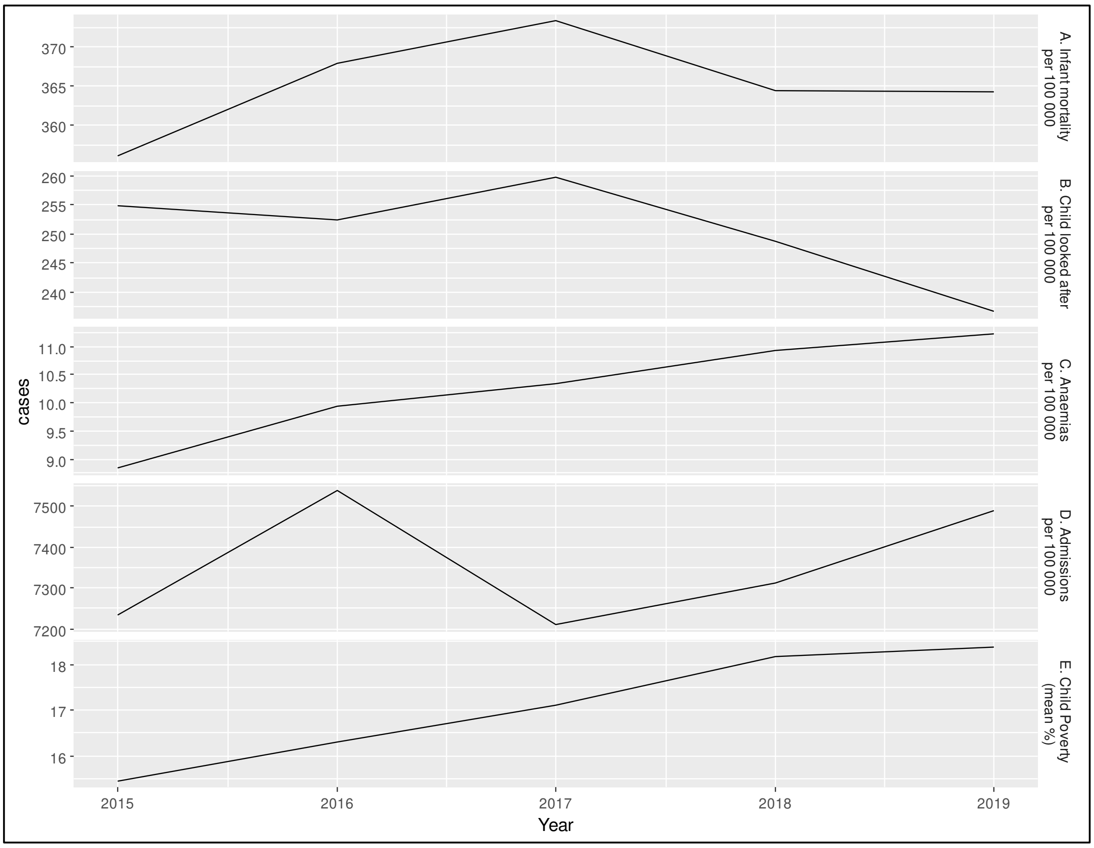

Figure A Annual trend in cases per 100 000 persons for each outcome measure and the population-weighted mean prevalence of relative child poverty before housing costs, 2015-2019 in England.

**Region**	Modelled change, *N* of events with 95% Confidence Intervals (CI)		
	Low ambition	Medium ambition	High ambition
Infant mortality			
**NATIONAL LEVEL**	-155<br>(-244, -62)	-224<br>(-353, -90)	-293<br>(-461, -118)
East Midlands	-10<br>(-16, -4)	-15<br>(-23, -6)	-19<br>(-30, -8)
East of England	-14<br>(-22, -6)	-20<br>(-31, -8)	-26<br>(-41, -10)
London	-29<br>(-46, -12)	-42<br>(-66, -17)	-55<br>(-86, -22)
North East	-9<br>(-15, -4)	-14<br>(-21, -5)	-18<br>(-28, -7)
North West	-25<br>(-39, -10)	-35<br>(-56, -14)	-46<br>(-73, -19)
South East	-16<br>(-25, -7)	-23<br>(-37, -9)	-31<br>(-48, -12)
South West	-9<br>(-14, -4)	-13<br>(-20, -5)	-17<br>(-27, -7)

West Midlands	-23<br>(-37, -9)	-34<br>(-53, -14)	-44<br>(-69, -18)
Yorkshire and the Humber	-20<br>(-31, -8)	-29<br>(-45, -11)	-37<br>(-59, -15)
Children looked after			
**NATIONAL LEVEL**	-2483<br>(-4015, -1051)	-3590<br>(-5804, -1519)	-4696<br>(-7593, -1987)
East Midlands	-167<br>(-270, -71)	-241<br>(-390, -102)	-316<br>(-511, -134)
East of England	-229<br>(-370, -97)	-331<br>(-535, -140)	-433<br>(-700, -183)
London	-415<br>(-672, -176)	-600<br>(-971, -254)	-786<br>(-1270, -332)
North East	-158<br>(-255, -67)	-228<br>(-368, -96)	-298<br>(-482, -126)
North West	-391<br>(-633, -166)	-565<br>(-914, -239)	-740<br>(-1196, -313)
South East	-275<br>(-444, -116)	-397<br>(-642, -168)	-520<br>(-840, -220)
South West	-154<br>(-249, -65)	-222<br>(-360, -94)	-291<br>(-470, -123)
West Midlands	-370<br>(-598, -156)	-534<br>(-864, -226)	-699<br>(-1131, -296)
Yorkshire and the Humber	-325<br>(-525, -137)	-469<br>(-759, -199)	-614<br>(-993, -260)
Hospitalisations for nutritional anaemias			
**NATIONAL LEVEL**	-242<br>(-304, -177)	-350<br>(-439, -257)	-458<br>(-574, -336)
East Midlands	-16<br>(-20, -12)	-24<br>(-30, -17)	-31<br>(-39, -23)
East of England	-22<br>(-28, -16)	-32<br>(-40, -23)	-42<br>(-53, -31)
London	-41<br>(-51, -30)	-59<br>(-74, -43)	-77<br>(-97, -56)
North East	-15<br>(-19, -11)	-22<br>(-28, -16)	-29<br>(-36, -21)
North West	-38<br>(-48, -28)	-55<br>(-69, -40)	-72<br>(-91, -53)
South East	-27<br>(-34, -20)	-39<br>(-49, -28)	-51<br>(-64, -37)
South West	-15<br>(-19, -11)	-22<br>(-27, -16)	-28<br>(-35, -21)
West Midlands	-36<br>(-45, -26)	-52<br>(-65, -38)	-68<br>(-86, -50)
Yorkshire and the Humber	-32<br>(-40, -23)	-46<br>(-57, -34)	-60<br>(-75, -44)
Emergency admissions			
**NATIONAL LEVEL**	-17 266<br>(-32 324, -2 127)	-24 958<br>(-46 725, -3 075)	-32 650<br>(-61 126, -4022)
East Midlands	-1161<br>(-2173, -143)	-1678<br>(-3142, -207)	-2195<br>(-4110, -270)
East of England	-1580	-2284	-2988

	(-2958, -195)	(-4276, -281)	(-5593, -368)
London	-2906<br>(-5440, -358)	-4200<br>(-7864, -517)	-5495<br>(-10287, -677)
North East	-1095<br>(-2050, -135)	-1583<br>(-2963, -195)	-2070<br>(-3876, -255)
North West	-2722<br>(-5097, -335)	-3935<br>(-7367, -485)	-5148<br>(-9638, -634)
South East	-1909<br>(-3573, -235)	-2759<br>(-5165, -340)	-3609<br>(-6757, -445)
South West	-1064<br>(-1992, -131)	-1538<br>(-2880, -190)	-2013<br>(-3768, -248)
West Midlands	-2571<br>(-4813, -317)	-3716<br>(-6957, -458)	-4861<br>(-9101, -599)
Yorkshire and the Humber	-2258<br>(-4228, -278)	-3265<br>(-6112, -402)	-4271<br>(-7996, -526)

Table A Estimated total change in events (95% Confidence Interval) for each scenario compared to baselined aggregated at national and regional levels between 2024 and 2033 from annual local authority changes across the period.

**IMD**	Modelled total change, *N* of events with 95% Confidence Intervals (CI)		
	Low ambition	Medium ambition	High ambition
Infant mortality			
1 (most deprived)	-67<br>(-105, -27)	-97<br>(-152, -39)	-126<br>(-199, -51)
2	-51<br>(-80, -20)	-73<br>(-115, -30)	-96<br>(-151, -39)
3 (least deprived)	-38<br>(-59, -15)	-54<br>(-85, -22)	-71<br>(-112, -29)
Children looked after			
1 (most deprived)	-1008<br>(-1630, -427)	-1457<br>(-2357, -617)	-1907<br>(-3083, -807)
2	-841<br>(-1360, -356)	-1216<br>(-1966, -514)	-1590<br>(-2572, -673)
3 (least deprived)	-634<br>(-1025, -268)	-916<br>(-1482, -388)	-1199<br>(-1939, -507)
Nutritional anaemia			
1 (most deprived)	-99<br>(-124, -72)	-143<br>(-179, -105)	-187<br>(-234, -137)
2	-82<br>(-103, -60)	-118<br>(-148, -87)	-155<br>(-194, -113)
3 (least deprived)	-62<br>(-77, -45)	-89<br>(-112, -65)	-117<br>(-146, -86)
Emergency admissions			
1 (most deprived)	-7034<br>(-13 169, -867)	-10 168<br>(-19 036, -1253)	-13 302<br>(-24 903, -1639)
2	-5830<br>(-10 916, -718)	-8428<br>(-15 779, -1038)	-11 025<br>(-20 642, -1358)
3 (least deprived)	-4401<br>(-8239, -542)	-6362<br>(-11 910, -784)	-8322<br>(-15 581, -1025)

Table B Estimated total change in events (95% Confidence Interval) for each scenario compared to baseline aggregated by deprivation level (IMD tertiles) between 2024 and 2033 from annual local authority changes across the period.

Relative change in inequalities<br>*Relative Index of Inequality (RII) Ratio, with 95% Confidence Intervals (CI)*		
Low	Medium	High
Infant mortality		
0.9940<br>(0.9906, 0.9976)	0.9914<br>(0.9864, 0.9965)	0.9887<br>(0.9822, 0.9955)
Children looked after		
0.9986<br>(0.9977, 0.9994)	0.9980<br>(0.9967, 0.9991)	0.9973<br>(0.9957, 0.9989)
Nutritional anaemias (ages <16)		
0.9970<br>(0.9962, 0.9978)	0.9956<br>(0.9945, 0.9968)	0.9942<br>(0.9927, 0.9958)
Emergency admissions (ages <16)		
0.9980<br>(0.9963, 0.9998)	0.9971<br>(0.9947, 0.9996)	0.9963<br>(0.9930, 0.9995)

Table C Change in the Relative Index of Inequality (RII) – RII ratio – under three child poverty reduction scenarios between 2024 and 2033, relative to a baseline scenario of increasing child poverty with 95% Confidence Intervals (CIs).

References

1. Moreno-Betancur M, Latouche A, Menvielle G, Kunst AE, Rey G. Relative Index of Inequality and Slope Index of Inequality. Epidemiology [Internet]. 2015 Jul;26(4):518–27. Available from: http://journals.lww.com/00001648-201507000-00012

2. Kypridemos C, Guzman-Castillo M, Hyseni L, Hickey GL, Bandosz P, Buchan I, et al. Estimated reductions in cardiovascular and gastric cancer disease burden through salt policies in England: an IMPACT NCD microsimulation study. BMJ Open [Internet]. 2017 Jan 24;7(1):e013791. Available from: https://bmjopen.bmj.com/lookup/doi/10.1136/bmjopen-2016-013791
